# Strengthening mental health care systems for Syrian refugees in Europe and the Middle East: integrating scalable psychological interventions in eight countries

**DOI:** 10.1080/20008198.2017.1388102

**Published:** 2017-11-07

**Authors:** Marit Sijbrandij, Ceren Acarturk, Martha Bird, Richard A Bryant, Sebastian Burchert, Kenneth Carswell, Joop de Jong, Cecilie Dinesen, Katie S. Dawson, Rabih El Chammay, Linde van Ittersum, Mark Jordans, Christine Knaevelsrud, David McDaid, Kenneth Miller, Naser Morina, A-La Park, Bayard Roberts, Yvette van Son, Egbert Sondorp, Monique C. Pfaltz, Leontien Ruttenberg, Matthis Schick, Ulrich Schnyder, Mark van Ommeren, Peter Ventevogel, Inka Weissbecker, Erica Weitz, Nana Wiedemann, Claire Whitney, Pim Cuijpers

**Affiliations:** ^a^ Clinical, Neuro and Developmental Psychology, VU University, Amsterdam, the Netherlands; ^b^ Department of Psychology, Istanbul Sehir University, Istanbul, Turkey; ^c^ International Federation of Red Cross and Red Crescent Societies Reference Centre for Psychosocial Support, Copenhagen, Denmark; ^d^ School of Psychology, University of New South Wales, Sydney, Australia; ^e^ Department of Clinical Psychological Intervention, Freie Universität Berlin, Berlin, Germany; ^f^ Department of Mental Health and Substance Abuse, World Health Organization, Geneva, Switzerland; ^g^ Faculty of Social and Behavioural Sciences, University of Amsterdam, Amsterdam, the Netherlands; ^h^ Ministry of Public Health, Beirut, Lebanon; ^i^ Department of Psychiatry, Faculty of Medicine, Saint Joseph University, Beirut, Lebanon; ^j^ Grants Desk, VU University Medical Center, Amsterdam, the Netherlands; ^k^ Department of Research and Development, War Child, Amsterdam, the Netherlands; ^l^ Center for Global Mental Health, Institute of Psychiatry, Psychology and Neuroscience, King’s College London, London, UK; ^m^ University Hospital Zurich, University of Zurich, Zurich, Switzerland; ^n^ Department of Health Policy, Personal Social Services Research Unit, London School of Economics and Political Science, London, UK; ^o^ Department of Health Services Research and Policy, London School of Hygiene and Tropical Medicine, London, UK; ^p^ Region Netherlands Centre and North, i-Psy Mental Health Care, Almere, the Netherlands; ^q^ KIT Royal Tropical Institute, Amsterdam, the Netherlands; ^r^ War Trauma Foundation, Diemen, the Netherlands; ^s^ Public Health Section, United Nations High Commissioner for Refugees, Geneva, Switzerland; ^t^ International Medical Corps, London, UK

**Keywords:** Refugees, Syria, psychological interventions, implementation, task-shifting, common mental disorders, cognitive behavioural therapy (CBT), problem solving treatment (PST), e-mental health interventions, Refugiados, Siria, intervenciones psicológicas, implementación, cambio de tareas, trastornos mentales comunes, terapia cognitivo-conductual (TCC), tratamiento de resolución de problemas (PST), intervenciones electrónicas de salud mental, 难民, 叙利亚, 心理干预, 执行, 任务切换, 常见心理障碍, 认知行为疗法（CBT）, 问题解决疗法（PST）, 网络心理健康干预, • Syrian refugees are at risk of developing common mental disorders, including depression and posttraumatic stress disorder. • Evidence-based interventions for refugees are available, but refugees have limited access to mental health services for these problems because of limited availability of mental health professionals in Europe and the Middle East. • STRENGTHS will translate and adapt a scalable set of World Health Organization interventions including the evidence-based PM+ for use with Syrian refugees with elevated levels of distress and reduced functioning.• The programmes will be delivered in individual, group or smartphone formats and will be supported by either peer-refugees or local non-professional helpers who will receive training and supervision.

## Abstract

The crisis in Syria has resulted in vast numbers of refugees seeking asylum in Syria’s neighbouring countries as well as in Europe. Refugees are at considerable risk of developing common mental disorders, including depression, anxiety, and posttraumatic stress disorder (PTSD). Most refugees do not have access to mental health services for these problems because of multiple barriers in national and refugee specific health systems, including limited availability of mental health professionals. To counter some of challenges arising from limited mental health system capacity the World Health Organization (WHO) has developed a range of scalable psychological interventions aimed at reducing psychological distress and improving functioning in people living in communities affected by adversity. These interventions, including Problem Management Plus (PM+) and its variants, are intended to be delivered through individual or group face-to-face or smartphone formats by lay, non-professional people who have not received specialized mental health training,

We provide an evidence-based rationale for the use of the scalable PM+ oriented programmes being adapted for Syrian refugees and provide information on the newly launched STRENGTHS programme for adapting, testing and scaling up of PM+ in various modalities in both neighbouring and European countries hosting Syrian refugees.

## Abstract (es)

La crisis en Siria ha dado lugar a un gran número de refugiados que buscan asilo en países vecinos a Siria, así como en Europa. Los refugiados corren un riesgo considerable de desarrollar trastornos mentales comunes, como depresión, ansiedad y trastorno por estrés postraumático (TEPT). La mayoría de los refugiados no tienen acceso a servicios de salud mental para estos problemas debido a las múltiples barreras existentes en los sistemas de salud nacionales y específicos para refugiados, incluida una limitada disponibilidad de profesionales de salud mental. Para contrarrestar algunos de los retos derivados de la limitada capacidad del sistema de salud mental, la Organización Mundial de la Salud (OMS) ha desarrollado una gama de intervenciones psicológicas escalables dirigidas a reducir la angustia psicológica y mejorar el funcionamiento de las personas afectadas por la adversidad. Estas intervenciones, que incluyen *Problem Management Plus* (Gestión de problemas plus, PM+) y sus variantes, están pensadas para ser aplicadas en formatos cara a cara o mediante teléfonos inteligentes a individuos o grupos por personas no profesionales que no han recibido formación especializada en salud mental,

Proporcionamos una justificación basada en la evidencia para el uso de programas escalables orientados a la PM+ que están siendo adaptados para refugiados sirios y proporcionamos información sobre el programa STRENGTHS recientemente lanzado para adaptar, probar y ampliar la PM+ en diversas modalidades, tanto en los países vecinos como en los europeos que reciben refugiados de Siria.

## Abstract (zh)

叙利亚危机导致了海量的难民到周边国家和欧洲寻求避难所。难民们发展出常见心理障碍的风险非常大，其中包括抑郁，焦虑，创伤后应激障碍（PTSD）。大多数难民没有渠道向心理健康机构寻求帮助，这主要是因为国家医疗和难民健康系统的种种阻碍，包括心理健康专家的数量不足。为了应对心理健康系统容量有限所产生的问题，世界卫生组织（WHO）发展出了一系列的可量化心理干预方法，用来减少心理障碍和提高社区中处于逆境里的人们的生活功能。这些干预方法中就有《问题应对量表Plus》（PM+）和它的其他变型，用来给未经过专业心理健康训练的非专业新手以面对面的或智能手机的形式向个人或群体使用。我们为针对叙利亚难民改良的可量化的PM+为基础的项目提供了有证据支持的原理，并且提供了关于新近运行的STRENGTHS项目的信息，该项目旨在在接受叙利亚难民的周边国家和欧洲国家中适应、测试、扩大PM+的多种形式。

## 1. Introduction

The armed conflict that has afflicted Syria since 2011 has resulted in a massive forced displacement of the Syrian population. In April 2017, there were approximately five million registered Syrian refugees (UNHCR, 2017). The majority of Syrian refugees have fled to Syria’s neighbouring countries. Turkey now hosts about three million Syrian refugees, Lebanon one million, and Jordan about 650,000 (UNHCR, 2017). Reports state that over 50% of Syrian refugees are children, in many cases unaccompanied by their families (UNICEF, 2016).

Syrian refugees may have been exposed to multiple war-related stressors such as torture, rape, witnessing the death of family members as well as the destruction of their homes and livelihoods, and they have undertaken a risky and stressful flight leaving their homeland for an unknown future (Silove, Ventevogel, & Rees, 2017). In addition to experiences of major loss and potentially traumatic experiences in their country of origin, Syrian refugees are also affected by stressful circumstances in host countries, where the capacity for self-help and mutual support has been negatively impacted by forced migration, the separation from families and communities, collective violence and mistrust. Poverty among Syrians living in Jordan, Lebanon and Turkey is widespread (Budosan, Aziz, Benner, & Abras, 2016), and their civil and employment rights are often limited. For example, Syrians in Jordan live in camp settings or overcrowded houses, relying in part on financial support from non-governmental organisations and have difficulties accessing jobs due to employment restrictions and livelihood opportunities (Gammouh, Al-Smadi, Tawalbeh, & Khoury, 2015). Many refugees, especially children and women, are vulnerable to exploitation, social isolation, gender-based violence or early marriage (World Bank, 2016; Boswall & Akash, 2015; Wells, Steel, Abo-Hilal, Hassan, & Lawsin, 2016). Finally, complicated registration processes hamper access to educational institutions and healthcare (Wells et al., 2016).

The impacts of refugee status are also challenging for refugees hosted within high-income western European countries. Post-migration stressors that refugees may face upon arrival in such European countries are to some extent similar to those in the countries surrounding Syria. They include cultural integration issues, the loss of family and community support, discrimination and adverse political climate, loneliness and boredom, prohibition to work, and disruption of education for children (Kirmayer et al., 2011; Miller & Rasmussen, 2010). In addition, uncertainties around the length of the asylum procedure, multiple dislocations, and the lack of recognition of degrees and other qualifications may increase levels of stress and discomfort in Syrian refugees living in high-income countries in Europe.

## 2. Common mental disorders and related conditions in Syrian refugees

Refugees are at considerable risk of developing symptoms of common mental disorders including depression, anxiety, posttraumatic stress disorder (PTSD) and related somatic health symptoms (de Jong, Komproe, & Van Ommeren, 2003; Fazel, Wheeler, & Danesh, 2005; Hassan, Ventevogel, Jefee-Bahloul, Barkil-Oteo, & Kirmayer, 2016; Steel et al., 2009). Epidemiological studies indicate that the age-standardized point prevalence of PTSD and major depression in conflict-affected populations is estimated to be 12.9% and 7.6%, respectively (Charlson et al., 2016). As a comparison, it has been estimated that approximately 4.4% of the world population suffers from major depression (WHO, 2017) and 3.3% from PTSD (Stein et al., 2014). Although good epidemiological data on psychosis is lacking, it is also likely that psychotic symptoms in Syrians have increased (Hassan et al., 2016; Hijazi & Weissbecker, 2015). Child refugees may be especially at risk of developing emotional and behavioural problems, with one study of Syrian refugee children in Turkey reporting that nearly half show clinically significant levels of anxiety and withdrawal (Cartwright, El-Khani, Subryan, & Calam, 2015).

Mental health problems are also relevant for social integration. For example, among refugees living in Switzerland a lack of social integration has been highly correlated with decreased health-related quality of life, functional impairment, and severity of depression and anxiety symptoms and symptoms of PTSD. Additionally, symptoms of PTSD and depression predicted difficulties in integration (Schick et al., 2016).

## 3. Mental health care for refugees across Europe and the Middle East

Current crises in the Middle East, such as the Syrian crisis, differ from many other large-scale displacements in previous decades in that a significant majority of displaced persons do not live in refugee camps. Instead people have settled in cities, towns, and villages in neighbouring countries such as Turkey, Lebanon, Jordan and Iraq, which creates new challenges for humanitarian actors in providing services. In these countries, the mental health services required to meet the demands of millions of refugees in need are inadequate and their health systems are overburdened to meet even basic survival needs as well as more chronic health problems (Gornall, 2015). Government expenditure on mental health as a percentage of total government health budgets in seven Arab countries ranged from just 2% in Syria and Egypt to 5% in Lebanon compared with approximately 11% in Germany and the Netherlands (WHO, 2011; Yehia, Nahas, & Saleh, 2014). In Jordan and Turkey, refugees are eligible to receive free access to mental health care facilities. In Middle Eastern countries, mental health care is largely confined to specialized psychiatric services for people with severe mental disorders (Alatas, Karaoglan, Arslan, & Yanik, 2009; Al-Krenawi, 2005). In addition to government health care services, international organizations and non-governmental organizations (NGOs) provide humanitarian support to refugees, such as cash, shelter, food, water, sanitation, and health services (Abo-Hilal & Hoogstad, 2013; El Chammay, Kheir, & Alaouie, 2013).

Within Europe, countries differ with respect to the degree that they provide access to healthcare for specific groups of migrants who have not yet been recognized as refugees, such as asylum seekers or undocumented migrants (Mladovsky, Rechel, Ingleby, & McKee, 2012). In Germany, asylum seekers and migrants have the right to attend state- or health insurance-funded psychotherapy, although administrative and practical barriers hamper access (Bozorgmehr, Razum, & Caylà, 2015). Other structural barriers to access to mental health care for refugees in European health care systems may include gatekeeping mechanisms that mean that a referral from primary care professionals such as general practitioners (GPs) is required for access to most secondary care services (OECD/EU, 2016). The level and extent of training for primary care professionals in both mental and refugee health will influence care pathways and access to care (Jensen, Norredam, Priebe, & Krasnik, 2013). Even in countries with considerable mental health services, the lack of Arabic-speaking health providers and interpreter services often hinders access to appropriate mental health care.

## 4. Evidence-based interventions for refugees

Although interventions that are effective in high-resource settings have also been shown to be effective in low-resource settings and for migrant populations (Morina, Malek, Nickerson, & Bryant, 2017), cultural adaptation of the original intervention protocols to the local culture is essential. During cultural adaptation, the intervention protocol is systematically modified considering language, culture, and context compatible with the client’s cultural meanings and values. The degree of such adaptation indeed proved to be associated with higher efficacy among guided self-help interventions (Harper Shehadeh, Heim, Chowdhary, Maercker, & Albanese, 2016).

European mental health care services generally offer psychotherapist or psychiatrist delivered, specialized mental health services that may involve a wide range of treatments, such as Cognitive Behavioural Therapy (CBT), Narrative Exposure Therapy (NET) (Stenmark, Catani, Neuner, Elbert, & Holen, 2013), Eye Movement Desensitization and Reprocessing (EMDR) (Ter Heide, Mooren, Kleijn, de Jongh, & Kleber, 2011), and individual-based multimodal interventions for PTSD (see Nickerson, Bryant, Silove, & Steel, 2011; Nose et al., 2017; Slobodin & de Jong, 2015; van Wyk & Schweitzer, 2014 for reviews) in refugees and asylum seekers. With respect to evidence for such interventions, a recent meta-analysis that examined psychological interventions for symptoms of PTSD in refugees and asylum seekers resettled in high-income countries identified 14 randomized controlled trials (RCTs) (Nose et al., 2017). It was shown that these interventions, mostly NET and CBT, were effective in reducing symptoms of PTSD and depression, with the strongest evidence base being for NET (Nose et al., 2017).

Treatment studies carried out with Syrian refugees, in particular, are being conducted and some have already been published. Two small RCTs in Syrian refugees located in Kilis refugee camp in Turkey evaluated the efficacy of EMDR for PTSD (Acarturk et al., 2015, 2016). These studies showed that both symptoms of PTSD and depression significantly reduced in refugees who received EMDR (Acarturk et al., 2015, 2016).

## 5. Barriers to mental health care for refugees

Numerous barriers to the delivery and uptake of mental health and psychosocial support interventions for refugee populations both in high-income Europe and countries surrounding Syria have been described.

Firstly, evidence-based interventions such as CBT, NET and EMDR are usually delivered by highly trained specialist mental health care providers. However, there are insufficient numbers of mental health care professionals to cover the needs of refugees experiencing impairing psychological distress.

Within Europe the large majority (80–90%) of refugees with symptoms of PTSD or other psychological problems (Laban, Gernaat, Komproe, & De Jong, 2007; Lamkaddem et al., 2014) do not visit specialized mental health care services. Other barriers include language problems (Bischoff et al., 2003), physical distance to mental health care services since refugees are often located in rural areas, and an overall lack of Arabic speaking psychologists or psychotherapists in European countries hosting Syrian refugees. In the Netherlands and Germany, waitlists for specialized mental health care for refugees of six months on average have been reported as a result of the recent increase in refugees (personal communication; i-Psy 2017; BAfF, 2016). In Germany, it has been estimated that of 379,848 refugees in need of mental health care in 2015, only 19,472 received treatment (about 5%; BAfF, 2016). In addition, the use of professional interpreters is expensive, and is generally perceived by migrants as hindering their treatment (Hadziabdic, Heikkilä, Albin, & Hjelm, 2009). Using interpreters from the network of the person, such as family members, can be problematic in the context of psychosocial interventions because of confidentiality and issues around potential vicarious traumatization especially when children are asked to be interpreters. Further, refugees themselves often lack knowledge about existing treatment possibilities (Maier & Straub, 2011), which may be the result of a lack of culturally appropriate information about the available services (Fassaert et al., 2009). Refugees, in common with many in the general population, may distrust mental health care, or avoid visiting mental health care practitioners because of shame, embarrassment or fear of rejection by family or friends and being labelled ‘mad’ or ‘crazy’ (Hassan et al., 2016).

## 6. Scaling-up mental health interventions for refugees

Major external events, such as conflict or disaster, usually challenge health systems’ capacity to adequately respond to the needs of vulnerable individuals and communities affected by these events (WHO, 2012). Ideally, health systems should be capable of quickly reacting to such external threats, in order to identify and adequately respond to the needs of large populations in need of health care. However, in reality health systems are not always well-equipped to deal with such situations. With respect to the delivery of evidence-based mental health care interventions to large numbers of refugees displaced as a result of the crisis in Syria, fundamentally there is a lack of human resources ready to deliver these interventions.

In 2008, the World Health Organization (WHO) launched the mental health Gap Action Programme (mhGAP) with a focus on low and middle income countries, including Jordan and Lebanon, with the goal of providing effective mental health treatments through primary and community care (WHO, 2010). A specific recommendation of WHO to increase the utilization and coverage of mental health care interventions in under-resourced settings is to implement task-shifting (or task-sharing) (WHO, 2010). Task-shifting means that a task that is originally performed by a highly-qualified specialist is transferred to a less specialized worker with fewer qualifications. For example, tasks may be shifted to a supervised lay person who is specifically trained to perform a limited task only. Through shifting tasks, interventions that are originally carried out in specialized services may be carried out in primary or community care instead (van Ginneken et al., 2013). In both high-resource European countries and Syrian neighbouring countries with fewer health care resources, task-shifting is a promising strategy to implement within current stepped care or collaborative care approaches to public health (de Jong, 2011; Thornicroft & Tansella, 2013). The more widespread availability of evidence-based task-shifting interventions for common mental disorders indirectly may also have a positive impact on the treatment gap for severe psychiatric disorders and associated symptoms problems such as psychosis and suicidal behaviours in LMICs. Task-shifting should allow for a more efficient allocation of the existing, albeit limited, specialist mental health staff and resources in any mental health care system towards the management of more severe psychiatric disorders.

Studies evaluating task-shifting interventions in mental health care have been carried out in LMICs, and show positive results in terms of reducing disability and improving overall and social functioning (Singla et al., 2017; van Ginneken et al., 2013). An RCT in India (Patel et al., 2010) showed that a collaborative care intervention led by lay counsellors was cost-saving to the health system (Buttorff et al., 2012). A 6–8 session behavioral activation treatment delivered by lay counsellors in primary health care settings in India was also cost effective, reducing depression and improving functioning in people with moderately severe to severe depression (Patel et al., 2017; Weobong et al., 2017). A systematic review of task-shifting interventions for non-communicable diseases in LMICs showed that it is potentially effective for improving access for mental healthcare (Joshi et al., 2014).

Task-shifting is also applicable to European mental health systems. European guidelines recommend stepped-care and collaborative care models as cost-effective alternatives to conventional care for common mental health symptoms in adults (Clark, 2011). Such stepped-care models may also be promising for scaling-up interventions for Syrian refugees by implementing shorter versions of regular CBT and/or problem-solving treatment (PST) or their e-mental health variants, as the first intervention before stepping up to more specialized treatments if these are indicated. These shorter and simpler first-step interventions may be delivered by trained lay-counsellors. However, as far as we know, stepped care models have not yet been implemented widely within European *refugee* mental health care. In the Netherlands, a preventive psychosocial task-shifting intervention is currently delivered by peer-refugees in Dutch asylum centers (Kieft, Jordans, de Jong, & Kamperman, 2008), but no studies evaluating the effects of the intervention have been carried out.

Challenges of task-shifting have also been described and include the need for an intensive training and supervision system, a lack of facilities (e.g. private space in primary health care centres), and high drop-out rates when task shifting is applied to volunteer lay helpers (Murray et al., 2014). Other barriers that have been described are insufficient contextual adaption of the methods (Hinton & Jalal, 2014), unfamiliarity with the materials, and practical difficulties in integrating new techniques within routine practice (Ventevogel & Spiegel, 2015). Finally, there is a need to identify barriers for successful large-scale implementation and dissemination of task-shifting interventions.

## 7. Scaling-up with e-mental health interventions

Another promising option for scaling-up mental health and psychosocial interventions within refugee populations is e-mental health interventions. E-mental health interventions may reach clients that would otherwise not have access to mental health treatment due to internal (e.g. fear of stigmatization) or external (e.g. infrastructure) barriers. Additional advantages are the relative brevity of e-mental health interventions and the possibility to automatize parts of the treatment. Both aspects increase the number of clients that can be treated by a single provider in a certain amount of time. This makes e-mental health interventions highly suitable for scenarios in which resources are limited or in which the capacities of traditional health systems do not suffice.

Until now, most e-mental health interventions evaluated are for classic website use. However, e-mental health interventions are now increasingly developed as smartphone e-mental health apps in order to increase their reach in populations affected by adversity (Ruzek, Kuhn, Jaworski, Owen, & Ramsey, 2016). These apps are especially promising for scaling-up in Syrian refugees since the majority of Syrians have access to mobile phones, and smartphones have become the main access point to the internet. A study in Za’atari refugee camp showed that approximately 90% of Syrians had a mobile phone, and 60% accessed the internet only through their smartphone (Maitland & Xu, 2015). Another advantage of mobile phone apps is that they can be used completely or partially offline which allows for better access to self-administered intervention tools in cases of unstable or unavailable internet access.

Within high resource settings, web-based e-mental health interventions for various psychiatric and somatic conditions (e.g. anxiety disorders, depression, body dissatisfaction, sexual dysfunction) have been meta-analytically shown to result in medium to large treatment effects that are comparable to those of their face-to-face equivalents (Andersson, Cuijpers, Carlbring, Riper, & Hedman, 2014). e-mental health interventions are usually based on CBT and they have been shown to be effective in reducing symptoms of common mental disorders such as PTSD (Kuester, Niemeyer, & Knaevelsrud, 2016; Sijbrandij, Kunovski, & Cuijpers, 2016), depression (Andrews, Cuijpers, Craske, McEvoy, & Titov, 2010), phobia (Andrews et al., 2010), panic disorder (Carlbring et al., 2006), and insomnia (van Straten et al., 2014), among others.

Until now, the evidence of e-mental health interventions in low resource settings is very limited. A recent study of Arabic-speaking individuals with PTSD comparing internet-delivered CBT to waitlist found significant reductions in symptoms of PTSD (Knaevelsrud, Brand, Lange, Ruwaard, & Wagner, 2015). Potential challenges of delivering e-mental health interventions to war-exposed populations such as Syrian refugees may include lack of trust in the political neutrality of a website or app and concerns about data storage, limits in confidential access to a device when a mobile phone may be shared among family members, the costs of internet or mobile use, and long-term sustainability of hosting and updating the interventions (Bockting, Williams, Carswell, & Grech, 2016).

## 8. Scaling up with the multi-component PM+ and related programmes

As part of its mhGAP programme, WHO is developing a range of scalable psychological interventions for use in settings affected by adversity. One of these, called Problem Management Plus (PM+), was designed for use in communities affected by adversity (Dawson et al., 2015). PM+ is available as an individual version (Individual PM+) evaluated in Pakistan (Rahman et al., 2016) and Kenya (Bryant et al., 2017) and as a group version (Group PM+) under evaluation in Swat, Pakistan (Chiumento et al., 2017; Khan et al., 2017).

These multi-behavioural interventions are short, for example the group and individual versions of PM+ are delivered over five weekly sessions of 90 minutes for the individual version and 120 minutes for the group version. They are transdiagnostic, since they address multiple mental health symptoms, rather than focusing explicitly on one disorder. They are multicomponent and based on evidence-based CBT and PST strategies. They may be delivered by non-professional helpers in community or primary care settings or by lay people such as peer-refugees after approximately 10 days of training followed by weekly group supervision by a trained clinician. Clients are taught four strategies: stress management (slow breathing exercises); problem solving (proactive management of practical difficulties through a series of sequential steps including selection of problems, brainstorming for solutions, planning implementation of solutions); behavioural activation (re-engaging with pleasant and task-oriented activities); and skills to strengthen one’s social support (see Dawson et al., 2015, for a more detailed description).

In a pilot RCT of 60 participants affected by terrorism and war in Peshawar, Pakistan (Rahman et al., 2016), the effect of PM+ Individual delivered by lay-counsellors was compared to enhanced treatment as usual (ETAU) consisting of management by primary care physicians with additional basic mental health training. PM+ Individual improved psychosocial functioning and reduced PTSD symptoms (Rahman et al., 2016). This study was followed by a large definitive RCT on PM+ Individual’s effectiveness in 346 individuals in the same area in Pakistan. This major study has shown that PM+ in Pakistan is effective as it is associated with greater improvements in anxiety, depression, functioning and posttraumatic stress than enhanced treatment as usual (Rahman et al., 2016).

## 9. The STRENGTHS programme

Addressing psychological distress and vulnerabilities is important to cope with the current refugee crisis and is a way to take into account the migrants’ long-term future beyond asylum requests. There are many challenges, however, in the detection, effective and cost effective delivery of evidence-based mental health programmes to Syrians suffering from distress related to loss, trauma and forced migration.

**Figure 1.**
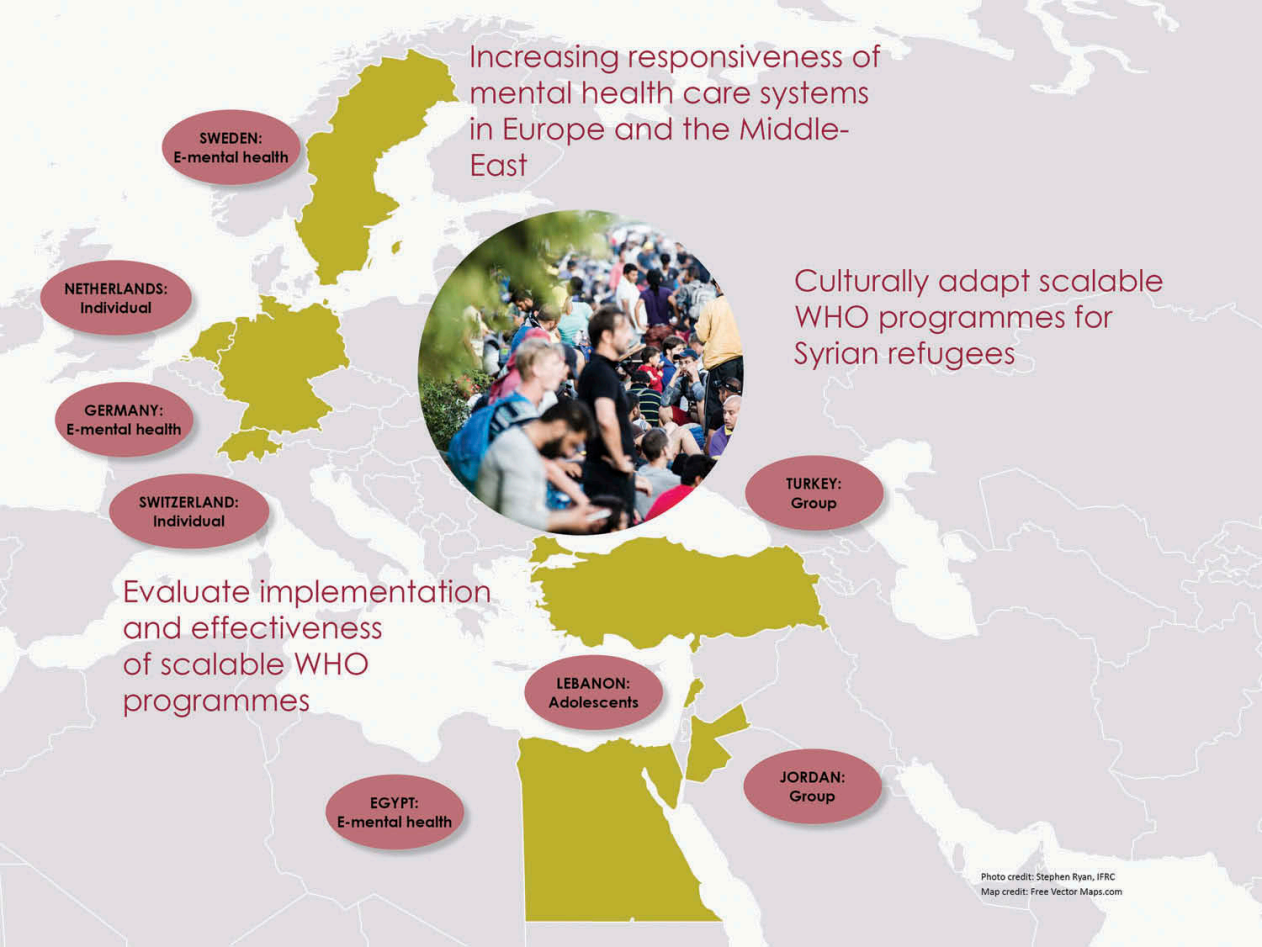
Overview of the STRENGTHS programme and implementation map.

The main goal of the EU STRENGTHS programme is to improve the responsiveness of mental health systems in Europe and key Middle Eastern countries by integrating mental health services for adult and adolescent Syrian refugees into primary and community care systems (Figure 1). STRENGTHS is coordinated by VU University Amsterdam, in the Netherlands, and includes academic and research institutions from Europe (Freie Universität Berlin, Istanbul Sehir University, KIT, London School of Economics and Political Science, London School of Hygiene and Tropical Medicine, University Hospital Zurich and the University of New South Wales), UN agencies (UNHCR), international agencies (International Medical Corps, the International Federation of Red Cross and Red Crescent Societies through its Reference Centre for Psychosocial Support), and NGOs and mental health care organizations such as War Child Holland, War Trauma Foundation, i-Psy Mental Health Care in the Netherlands and the ‘Mülteciler ve sığınmacılar yardımlaşma ve dayanışma derneği’ Organization in Istanbul, Turkey. The advisory board includes international experts on refugee mental health care and Syrian mental health professionals.

The EU Horizon2020 STRENGTHS programme will translate and adapt a scalable set of WHO interventions including PM+ for use with adult and child Syrian refugees. STRENGTHS will study the scale-up of these programmes for Syrian refugees with elevated levels of distress and reduced functioning. STRENGTHS will implement the PM+ group interventions in adult Syrian refugees in Jordan and Turkey, and the PM+ Individual versions in the Netherlands and Switzerland. In Lebanon, implementation of the newly developed scalable group intervention for young adolescents will be evaluated. The smartphone-based transdiagnostic programme will be implemented in adult refugees across Germany, Egypt and Sweden. The programmes will be supported by either peer-refugees or local non-professional helpers who will receive training and supervision from local mental health care professionals.

The overall goal of STRENGTHS is to evaluate whether implementation of the WHO programmes improves the functioning and responsiveness of mental health systems to refugees across Europe and countries bordering Syria. Necessary steps to effectively integrate the programmes into the various health systems of the participating project countries will be determined. We will translate and adapt the WHO interventions and training programmes for use with Syrian refugees, and implementation trials will evaluate their effectiveness and cost-effectiveness in terms of improved health outcomes (depression and anxiety), improved overall functioning, and reduced health costs. We will also assess implementation outcomes such as the process of recruiting and retaining staff, fidelity, reach, dose and quality of the delivered interventions. In addition, we will identify what is needed for scaling-up in terms of investments in money, workforce, organisation and political requirements across all project countries. STRENGTHS will also aggregate all data of the implementation trials to determine predictors for treatment outcome across the different interventions and target groups. Finally, the evidence-based WHO interventions and strategies for implementation will be disseminated to stakeholders across the project countries and beyond.

## 10. Conclusion

More than five years of violent conflict in Syria have left one-quarter to one-third of the Syrian population internally displaced or seeking refuge abroad. In addition to daily living difficulties, Syrian refugees have reported symptoms of anxiety, depression, anger, fear, and excessive stress affecting both their significant relationships and daily functioning.

The refugee crisis imposes challenging demands on health systems both across the countries bordering Syria and European countries. The most significant barriers to delivery of evidence-based mental health care interventions to Syrian refugees are the lack of mental health professionals, and the lack of scalable evidence-based interventions targeted at reducing general distress as a result of past and ongoing stressors in Syrians.

As a promising strategy to reduce prolonged disabling distress in Syrians, we propose that the evidence-based scalable WHO interventions are evaluated and integrated into primary and community care to reduce common mental disorders for refugees across Middle Eastern and European countries. These programmes, including PM+ and its variants, are developed as face-to-face versions for adult individuals and groups, for young adolescents and in a version for smartphone use. They can likely be delivered by trained lay-counsellors including peer-refugees. With the large-scale implementation of the PM+ programmes, the STRENGTHS programme aims to strengthen responsiveness of national and local health care systems affected by the Syrian refugee crisis and to significantly reduce the burden of disease among vulnerable people such as Syrian refugees affected by war and displacement. STRENGTHS also aims to provide insights and recommendations on effective implementation mechanisms to respond more rapidly to the needs of other contemporary and future populations affected by conflict.

## References

[CIT0001] Abo-Hilal M., & Hoogstad M. (2013). Syrian mental health professionals as refugees in Jordan: Establishing mental health services for fellow refugees. *Intervention*, 11(1), 89–11. doi:10.1097/WTF.0b013e32835f0d2c

[CIT0002] Acarturk C., Konuk E., Cetinkaya M., Senay I., Sijbrandij M., Cuijpers P., & Aker T. (2015). EMDR for Syrian refugees with posttraumatic stress disorder symptoms: Results of a pilot randomized controlled trial. *European Journal of Psychotraumatology*, 6, 27414. doi:10.3402/ejpt.v6.27414 25989952 PMC4438099

[CIT0003] Acarturk C., Konuk E., Cetinkaya M., Senay I., Sijbrandij M., Gulen B., & Cuijpers P. (2016). The efficacy of eye movement desensitization and reprocessing for post-traumatic stress disorder and depression among Syrian refugees: Results of a randomized controlled trial. *Psychological Medicine*, 46(12), 2583–2593. doi:10.1017/S0033291716001070 27353367

[CIT0004] Alatas G., Karaoglan A., Arslan M., & Yanik M. (2009). Community-based psychiatry model and project of community mental health centers in Turkey. *Noropsikiyatri Arsivi-Archives of Neuropsychiatry*, 46, 25–29.

[CIT0005] Al-Krenawi A. (2005). Mental health practice in Arab countries. *Current Opinion in Psychiatry*, 18(5), 560–564. doi:10.1097/01.yco.0000179498.46182.8b 16639119

[CIT0006] Andersson G., Cuijpers P., Carlbring P., Riper H., & Hedman E. (2014). Guided Internet-based vs. face-to-face cognitive behavior therapy for psychiatric and somatic disorders: A systematic review and meta-analysis. *World Psychiatry*, 13(3), 288–295. doi:10.1002/wps.20151 25273302 PMC4219070

[CIT0007] Andrews G., Cuijpers P., Craske M. G., McEvoy P., Titov N., & Baune B. T. (2010). Computer therapy for the anxiety and depressive disorders is effective, acceptable and practical health care: A meta-analysis. *PLoS One*, 5(10), e13196. doi:10.1371/journal.pone.0013196 20967242 PMC2954140

[CIT0008] BAfF (2016). *Versorgungsbericht Zur psychosozialen Versorgung von Flüchtlingen und Folteropfern in Deutschland. 3. aktualisierte Auflage*. Berlin, Germany: Bundesweite Arbeitsgemeinschaft derPsychosozialen Zentren für Flüchtlinge Retrieved from http://www.baff-zentren.org/wp-content/uploads/2017/02/Versorgungsbericht_3-Auflage_BAfF.pdf

[CIT0009] Bischoff A., Bovier P. A., Isah R., Françoise G., Ariel E., & Louis L. (2003). Language barriers between nurses and asylum seekers: Their impact on symptom reporting and referral. *Social Science & Medicine*, 57(3), 503–512. doi:10.1016/S0277-9536(02)00376-3 12791492

[CIT0010] Bockting C. L. H., Williams A. D., Carswell K., & Grech A. E. (2016). The potential of low-intensity and online interventions for depression in low- and middle-income countries. *Global Mental Health*, 3 ARTN e25. doi:10.1017/gmh.2016.21 PMC5454763 28596893

[CIT0011] Boswall K., & Akash R. A. (2015). Personal perspectives of protracted displacement: An ethnographic insight into the isolation and coping mechanisms of Syrian women and girls living as urban refugees in northern Jordan. *Intervention*, 13(3), 203–215. doi:10.1097/wtf.0000000000000097

[CIT0012] Bozorgmehr K., Razum O., & Caylà J. A. (2015). Effect of restricting access to health care on health expenditures among asylum-seekers and refugees: A quasi-experimental study in Germany, 1994–2013. *PLoS One*, 10(7), e0131483. doi:10.1371/journal.pone.0131483 26201017 PMC4511805

[CIT0013] Bryant R. A., Schafer A., Dawson K. S., Anjuri D., Mulili C., Ndogoni L., & van Ommeren M. (2017). Effectiveness of a brief behavioural intervention on psychological distress among women with a history of gender-based violence in urban Kenya: A randomised clinical trial. *PLoS Medicine*, 14(8), e1002371. doi:10.1371/journal.pmed.1002371 28809935 PMC5557357

[CIT0014] Budosan B., Aziz S., Benner M. T., & Abras B. (2016). Perceived needs and daily stressors in an urban refugee setting: Humanitarian Emergency Settings Perceived Needs Scale survey of Syrian refugees in Kilis, Turkey. *Intervention*, 14(3), 293–304. doi:10.1097/wtf.0000000000000123

[CIT0015] Buttorff C., Hock R. S., Weiss H. A., Naik S., Araya R., Kirkwood B. R., & Patel V. (2012). Economic evaluation of a task-shifting intervention for common mental disorders in India. *Bulletin of the World Health Organization*, 90(11), 813–821. doi:10.2471/BLT.12.104133 23226893 PMC3506405

[CIT0016] Carlbring P., Bohman S., Brunt S., Buhrman M., Westling B. E., Ekselius L., & Andersson G. (2006). Remote treatment of panic disorder: A randomized trial of internet-based cognitive behavior therapy supplemented with telephone calls. *The American Journal of Psychiatry*, 163(12), 2119–2125. doi:10.1176/ajp.2006.163.12.2119 17151163

[CIT0017] Cartwright K., El-Khani A., Subryan A., & Calam R. (2015). Establishing the feasibility of assessing the mental health of children displaced by the Syrian conflict. *Global Mental Health*, 2. doi:10.1017/gmh.2015.3 PMC5269638 28596856

[CIT0018] Charlson F. J., Flaxman A., Ferrari A. J., Vos T., Steel Z., & Whiteford H. A. (2016). Post-traumatic stress disorder and major depression in conflict-affected populations: An epidemiological model and predictor analysis. *Glob Mental Health (Camb)*, 3, e4. doi:10.1017/gmh.2015.26 PMC5314754 28596873

[CIT0019] Chiumento A., Hamdani S. U., Khan M. N., Dawson K., Bryant R. A., Sijbrandij M., & Rahman A. (2017). Evaluating effectiveness and cost-effectiveness of a group psychological intervention using cognitive behavioural strategies for women with common mental disorders in conflict-affected rural Pakistan: Study protocol for a randomised controlled trial. *Trials*, 18(1), 190. doi:10.1186/s13063-017-1905-8 28441974 PMC5405533

[CIT0020] Clark D. M. (2011). Implementing NICE guidelines for the psychological treatment of depression and anxiety disorders: The IAPT experience. *International Review of Psychiatry (Abingdon, England)*, 23(4), 318–327. doi:10.3109/09540261.2011.606803 PMC3212920 22026487

[CIT0021] Dawson K. S., Bryant R. A., Harper M., Kuowei Tay A., Rahman A., Schafer A., & van Ommeren M. (2015). Problem Management Plus (PM+): A WHO transdiagnostic psychological intervention for common mental health problems. *World Psychiatry*, 14(3), 354–357. doi:10.1002/wps.20255 26407793 PMC4592660

[CIT0022] de Jong J. (2011). (Disaster) Public mental health In Stein D., Friedman M., & Blanco C. (Eds.), *Post-traumatic stress disorder* (pp. 217–262). Hoboken, NJ: John Wiley & Sons.

[CIT0023] de Jong J. T., Komproe I. H., & Van Ommeren M. (2003). Common mental disorders in postconflict settings. *The Lancet*, 361(9375), 2128–2130. doi:10.1016/S0140-6736(03)13692-6 12826440

[CIT0024] El Chammay R., Kheir W., & Alaouie H. (2013). *Assessment of mental health and psychosocial support services for Syrian refugees in Lebanon*. Beirut: UNHCR Lebanon.

[CIT0025] Fassaert T., De Wit M. A. S., Tuinebreijer W. C., Verhoeff A. P., Beekman A. T. F., & Dekker J. (2009). Perceived need for mental health care among non-western labour migrants. *Social Psychiatry and Psychiatric Epidemiology*, 44(3), 208–216. doi:10.1007/s00127-008-0418-x 18787746

[CIT0026] Fazel M., Wheeler J., & Danesh J. (2005). Prevalence of serious mental disorder in 7000 refugees resettled in western countries: A systematic review. *The Lancet*, 365(9467), 1309–1314. doi:10.1016/S0140-6736(05)61027-6 15823380

[CIT0027] Gammouh O. S., Al-Smadi A. M., Tawalbeh L. I., & Khoury L. S. (2015). Chronic diseases, lack of medications, and depression among Syrian refugees in Jordan, 2013-2014. *Preventing Chronic Disease*, 12, E10. doi:10.5888/pcd12.140424 25633485 PMC4310712

[CIT0028] Gornall J. (2015). Healthcare for Syrian refugees. *BMJ*, 351, h4150. doi:10.1136/bmj.h4150 26243793

[CIT0029] Hadziabdic E., Heikkilä K., Albin B., & Hjelm K. (2009). Migrants’ perceptions of using interpreters in health care. *International Nursing Review*, 56(4), 461–469. doi:10.1111/j.1466-7657.2009.00738.x 19930075

[CIT0030] Harper Shehadeh M., Heim E., Chowdhary N., Maercker A., & Albanese E. (2016). Cultural Adaptation of Minimally Guided Interventions for Common Mental Disorders: A Systematic Review and Meta-Analysis. *JMIR Mental Health*, 3(3), e44. doi:10.2196/mental.5776 27670598 PMC5057065

[CIT0031] Hassan G., Ventevogel P., Jefee-Bahloul H., Barkil-Oteo A., & Kirmayer L. J. (2016). Mental health and psychosocial wellbeing of Syrians affected by armed conflict. *Epidemiology and Psychiatric Sciences*, 25(2), 129–141. doi:10.1017/S2045796016000044 26829998 PMC6998596

[CIT0032] Hijazi Z., & Weissbecker I. (2015). *Syria crisis. Addressing regional mental health needs and gaps in the context of the Syria crisis. International medical corps*. Washingron, DC: International Medical Corps Retrieved from http://internationalmedicalcorps.org/document.doc?id=526

[CIT0033] Hinton D. E., & Jalal B. (2014). Parameters for creating culturally sensitive CBT: Implementing CBT in global settings. *Cognitive and Behavioral Practice*, 21(2), 139–144. doi:10.1016/j.cbpra.2014.01.009

[CIT0034] Jensen N. K., Norredam M., Priebe S., & Krasnik A. (2013). How do general practitioners experience providing care to refugees with mental health problems? A qualitative study from Denmark. *BMC Family Practice*, 14, 17. doi:10.1186/1471-2296-14-17 23356401 PMC3568406

[CIT0035] Joshi R., Alim M., Kengne A. P., Jan S., Maulik P. K., Peiris D., & Patel A. A. (2014). Task shifting for non-communicable disease management in low and middle income countries - a systematic review. *PLoS One*, 9(8), ARTN e103754. doi:10.1371/journal.pone.0103754 PMC4133198 25121789

[CIT0036] Khan M. N., Hamdani S. U., Chiumento A., Dawson K., Bryant R. A., Sijbrandij M., & Rahman A. (2017). Evaluating feasibility and acceptability of a group WHO trans-diagnostic intervention for women with common mental disorders in rural Pakistan: A cluster randomised controlled feasibility trial. *Epidemiology and Psychiatric Sciences*, 1–11. doi:10.1017/S2045796017000336 PMC6998939 28689511

[CIT0037] Kieft B., Jordans M. J., de Jong J. T., & Kamperman A. M. (2008). Paraprofessional counselling within asylum seekers’ groups in the Netherlands: Transferring an approach for a non-western context to a European setting. *Transcultural Psychiatry*, 45(1), 105–120. doi:10.1177/1363461507088000 18344254

[CIT0038] Kirmayer L. J., Narasiah L., Munoz M., Rashid M., Ryder A. G., Guzder J., & Refugee H. (2011). Common mental health problems in immigrants and refugees: General approach in primary care. *CMAJ*, 183(12), E959–967. doi:10.1503/cmaj.090292 20603342 PMC3168672

[CIT0039] Knaevelsrud C., Brand J., Lange A., Ruwaard J., & Wagner B. (2015). Web-based psychotherapy for posttraumatic stress disorder in war-traumatized Arab patients: Randomized controlled trial. *Journal of Medical Internet Research*, 17(3), e71. doi:10.2196/jmir.3582 25799024 PMC4385175

[CIT0040] Kuester A., Niemeyer H., & Knaevelsrud C. (2016). Internet-based interventions for posttraumatic stress: A meta-analysis of randomized controlled trials. *Clinical Psychology Review*, 43, 1–16. doi:10.1016/j.cpr.2015.11.004 26655959

[CIT0041] Laban C. J., Gernaat H. B., Komproe I. H., & De Jong J. T. (2007). Prevalence and predictors of health service use among Iraqi asylum seekers in the Netherlands. *Social Psychiatry and Psychiatric Epidemiology*, 42(10), 837–844. doi:10.1007/s00127-007-0240-x 17676250 PMC2039804

[CIT0042] Lamkaddem M., Stronks K., Devillé W. D., Olff M., Gerritsen A. A., & Essink-Bot M.-L. (2014). Course of post-traumatic stress disorder and health care utilisation among resettled refugees in the Netherlands. *BMC Psychiatry*, 14, 90. doi:10.1186/1471-244X-14-90 24670251 PMC3986925

[CIT0043] Maier T., & Straub M. (2011). “My head is like a bag full of rubbish”: Concepts of illness and treatment expectations in traumatized migrants. *Qualitative Health Research*, 21(2), 233–248. doi:10.1177/1049732310383867 20876548

[CIT0044] Maitland C., & Xu Y. (2015). *A social informatics analysis of refugee mobile phone use: A case study of Zaaatari Syrian refugee camp* . Paper presented at the TPRC 43: The 43rd Research Conference on Communication, Information and Internet Policy Paper SSRN Electronic Journal doi:10.2139/ssrn.2588300

[CIT0045] Miller K. E., & Rasmussen A. (2010). War exposure, daily stressors, and mental health in conflict and post-conflict settings: Bridging the divide between trauma-focused and psychosocial frameworks. *Social Science & Medicine (1982)*, 70(1), 7–16. doi:10.1016/j.socscimed.2009.09.029 19854552

[CIT0046] Mladovsky P., Rechel B., Ingleby D., & McKee M. (2012). Responding to diversity: An exploratory study of migrant health policies in Europe. *Health Policy*, 105(1), 1–9. doi:10.1016/j.healthpol.2012.01.007 22306024

[CIT0047] Morina N., Malek M., Nickerson A., & Bryant R. A. (2017). Meta-analysis of interventions for posttraumatic stress disorder and depression in adult survivors of mass violence in low- and middle-income countries. *Depression and Anxiety*, 34, 679–691. doi:10.1002/da.22618 28419625

[CIT0048] Murray L. K., Tol W., Jordans M., Sabir G., Amin A. M., Bolton P., & Thornicroft G. (2014). Dissemination and implementation of evidence based, mental health interventions in post conflict, low resource settings. *Intervention*, 12, 94–115. doi:10.1097/WTF.0000000000000070 28316559 PMC5356225

[CIT0049] Nickerson A., Bryant R. A., Silove D., & Steel Z. (2011). A critical review of psychological treatments of posttraumatic stress disorder in refugees. *Clinical Psychology Review*, 31(3), 399–417. doi:10.1016/j.cpr.2010.10.004 21112681

[CIT0050] Nose M., Ballette F., Bighelli I., Turrini G., Purgato M., Tol W., & Barbui C. (2017). Psychosocial interventions for post-traumatic stress disorder in refugees and asylum seekers resettled in high-income countries: Systematic review and meta-analysis. *PLoS One*, 12(2), e0171030. doi:10.1371/journal.pone.0171030 28151992 PMC5289495

[CIT0051] OECD/EU (2016). Chapter 2. Strengthening primary care systems In Eu O. (Ed.), *Health at a glance: Europe 2016*. Paris: OECD Publishing.

[CIT0052] Patel V., Weiss H. A., Chowdhary N., Naik S., Pednekar S., Chatterjee S., & Kirkwood B. R. (2010). Effectiveness of an intervention led by lay health counsellors for depressive and anxiety disorders in primary care in Goa, India (MANAS): A cluster randomised controlled trial. *The Lancet*, 376(9758), 2086–2095. doi:10.1016/S0140-6736(10)61508-5 PMC4964905 21159375

[CIT0053] Patel V., Weobong B., Weiss H. A., Anand A., Bhat B., Katti B., & Fairburn C. G. (2017). The Healthy Activity Program (HAP), a lay counsellor-delivered brief psychological treatment for severe depression, in primary care in India: A randomised controlled trial. *The Lancet*, 389(10065), 176–185. doi:10.1016/S0140-6736(16)31589-6 PMC5236064 27988143

[CIT0054] Rahman A., Hamdani S. U., Awan N. R., Bryant R. A., Dawson K. S., Khan M. F., & van Ommeren M. (2016). Effect of a multicomponent behavioral intervention in adults impaired by psychological distress in a conflict-affected area of Pakistan: A randomized clinical trial. *JAMA*, 316(24), 2609–2617. doi:10.1001/jama.2016.17165 27837602

[CIT0055] Rahman A., Riaz N., Dawson K. S., Usman Hamdani S., Chiumento A., Sijbrandij M., & Farooq S. (2016). Problem Management Plus (PM+): Pilot trial of a WHO transdiagnostic psychological intervention in conflict-affected Pakistan. *World Psychiatry*, 15(2), 182–183. doi:10.1002/wps.20312 27265713 PMC4911784

[CIT0056] Ruzek J. I., Kuhn E., Jaworski B. K., Owen J. E., & Ramsey K. M. (2016). Mobile mental health interventions following war and disaster. *Mhealth*, 2, 37. doi:10.21037/mhealth.2016.08.06 28293610 PMC5344166

[CIT0057] Schick M., Zumwald A., Knöpfli B., Nickerson A., Bryant R. A., Schnyder U., & Morina N. (2016). Challenging future, challenging past: The relationship of social integration and psychological impairment in traumatized refugees. *European Journal of Psychotraumatology*, 7, 28057. doi:10.3402/ejpt.v7.28057 26886484 PMC4756625

[CIT0058] Sijbrandij M., Kunovski I., & Cuijpers P. (2016). Effectiveness of internet-delivered cognitive behavioral therapy for posttraumatic stress disorder: A systematic review and meta-analysis. *Depression and Anxiety*, 33(9), 783–791. doi:10.1002/da.22533 27322710

[CIT0059] Silove D., Ventevogel P., & Rees S. (2017). *The contemporary refugee crisis: An overview of mental health challenges*. World Psychiatry. 10.1002/wps.20438 PMC5428192 28498581

[CIT0060] Singla D. R., Kohrt B., Murray L. K., Anand A., Chorpita B. F., & Patel V. (2017). Psychological treatments for the world: Lessons from low- and middle-income countries. *Annual Review of Clinical Psychology*, 13, 149–181. doi:10.1146/annurev-clinpsy-032816-045217 PMC5506549 28482687

[CIT0061] Slobodin O., & de Jong J. T. (2015). Mental health interventions for traumatized asylum seekers and refugees: What do we know about their efficacy? *The International Journal of Social Psychiatry*, 61(1), 17–26. doi:10.1177/0020764014535752 24869847

[CIT0062] Steel Z., Chey T., Silove D., Marnane C., Bryant R. A., & van Ommeren M. (2009). Association of torture and other potentially traumatic events with mental health outcomes among populations exposed to mass conflict and displacement: A systematic review and meta-analysis. *JAMA*, 302(5), 537–549. doi:10.1001/jama.2009.1132 19654388

[CIT0063] Stein D. J., McLaughlin K. A., Koenen K. C., Atwoli L., Friedman M. J., Hill E. D., & Kessler R. C. (2014). DSM-5 and ICD-11 definitions of posttraumatic stress disorder: Investigating “narrow” and “broad” approaches. *Depression and Anxiety*, 31(6), 494–505. doi:10.1002/da.22279 24894802 PMC4211431

[CIT0064] Stenmark H., Catani C., Neuner F., Elbert T., & Holen A. (2013). Treating PTSD in refugees and asylum seekers within the general health care system. A randomized controlled multicenter study. *Behaviour Research and Therapy*, 51(10), 641–647. doi:10.1016/j.brat.2013.07.002 23916633

[CIT0065] Ter Heide F. J., Mooren T. M., Kleijn W., de Jongh A., & Kleber R. J. (2011). EMDR versus stabilisation in traumatised asylum seekers and refugees: Results of a pilot study. *European Journal of Psychotraumatology*, 2, 5881. doi:10.3402/ejpt.v2i0.5881 PMC3402110 22893808

[CIT0066] Thornicroft G., & Tansella M. (2013). The balanced care model for global mental health. *Psychological Medicine*, 43(4), 849–863. doi:10.1017/S0033291712001420 22785067

[CIT0067] UNHCR (2017). Syria regional refugee response; Inter-agency information sharing portal. Retrieved from http://data.unhcr.org/syrianrefugees/regional.php

[CIT0068] UNICEF (2016). Syrian refugees and other affected populations in Egypt, Iraq, Jordan, Lebanon and Turkey. Retrieved from http://www.unicef.org/appeals/syrianrefugees.html

[CIT0069] van Ginneken N., Tharyan P., Lewin S., Rao G. N., Meera S. M., Pian J., & Patel V. (2013). Non-specialist health worker interventions for the care of mental, neurological and substance-abuse disorders in low- and middle-income countries. *Cochrane Database of Systematic Reviews (Online)*, 11, CD009149. doi:10.1002/14651858.CD009149.pub2 24249541

[CIT0070] van Straten A., Emmelkamp J., De Wit J., Lancee J., Andersson G., van Someren E. J., & Cuijpers P. (2014). Guided Internet-delivered cognitive behavioural treatment for insomnia: A randomized trial. *Psychological Medicine*, 44(7), 1521–1532. doi:10.1017/S0033291713002249 24001364

[CIT0071] van Wyk S., & Schweitzer R. D. (2014). A systematic review of naturalistic interventions in refugee populations. *Journal of Immigrant and Minority Health / Center for Minority Public Health*, 16(5), 968–977. doi:10.1007/s10903-013-9835-3 23666201

[CIT0072] Ventevogel P., & Spiegel P. (2015). Psychological treatments for orphans and vulnerable children affected by traumatic events and chronic adversity in Sub-Saharan Africa. *JAMA*, 314(5), 511–512. doi:10.1001/jama.2015.8383 26241602

[CIT0073] Wells R., Steel Z., Abo-Hilal M., Hassan A. H., & Lawsin C. (2016). Psychosocial concerns reported by Syrian refugees living in Jordan: Systematic review of unpublished needs assessments. *The British Journal of Psychiatry: The Journal of Mental Science*, 209(2), 99–106. doi:10.1192/bjp.bp.115.165084 27103679

[CIT0074] Weobong B., Weiss H. A., McDaid D., Singla D. R., Hollon S. D., Nadkarni A., & Patel V. (2017). Sustained effectiveness and cost-effectiveness of the Healthy Activity Programme, a brief psychological treatment for depression delivered by lay counsellors in primary care: 12-month follow-up of a randomised controlled trial. *PLoS Medicine*, 14(9), e1002385. doi:10.1371/journal.pmed.1002385 28898283 PMC5595303

[CIT0075] World Bank (2016). *The welfare of Syrian refugees; Evidence from Jordan and Lebanon*. Geneva: World Bank Retrieved from https://openknowledge.worldbank.org/bitstream/handle/10986/23228/9781464807701.pdf?sequence=21&isAllowed=y

[CIT0076] WHO (2010). *mhGAP intervention guide for mental, neurological and substance use disorders in non-specialized health settings*. Geneva, Switzerland: Author. 23741783

[CIT0077] WHO (2011). Mental health atlas-2011. Retrieved from http://www.who.int/mental_health/evidence/atlas/profiles/en/

[CIT0078] WHO (2012). *Strengthening health-system emergency preparedness: Toolkit for assessing health-system capacity for crisis management*. Geneva: WHO Retrieved from http://www.euro.who.int/__data/assets/pdf_file/0008/157886/e96187.pdf

[CIT0079] WHO (2017). *Depression and other common mental disorders; Global health estimates*. Geneva: World Health Organiziation.

[CIT0080] Yehia F., Nahas Z., & Saleh S. (2014). A roadmap to parity in mental health financing: The case of Lebanon. *The Journal of Mental Health Policy and Economics*, 17(3), 131–141. 25543116

